# Evaluation of psychometric properties and factorial structure of the pre-school child behaviour checklist at the Kenyan Coast

**DOI:** 10.1186/s13034-015-0089-9

**Published:** 2016-01-20

**Authors:** Symon M. Kariuki, Amina Abubakar, Elizabeth Murray, Alan Stein, Charles R. J. C. Newton

**Affiliations:** KEMRI-Wellcome Trust Collaborative Research Programme, PO Box 230 (80108), Kilifi, Kenya; Nuffield Department of Medicine, University of Oxford, Oxford, UK; Department of Psychology, Lancaster University, Lancaster, UK; Department of Psychiatry, University of Oxford, Oxford, UK

**Keywords:** Child Behaviour Checklist, Factor analysis, Psychometric properties, Preschool children, Kenya

## Abstract

**Background:**

Behavioural/emotional problems may be common in preschool children living in resource-poor settings, but assessment of these problems in preschool children from poor areas is challenging owing to lack of appropriate behavioural screening tools. The child behaviour checklist (CBCL) is widely known for its reliability in identifying behavioural/emotional problems in preschool children, but it has not been validated for use in sub-Saharan Africa.

**Methods:**

With permission from developers of CBCL, we translated this tool into Ki-Swahili and adapted the items to make them culturally appropriate and contextually relevant and examined the psychometric properties of the CBCL, particularly reliability, validity and factorial structure in a Kenyan community preschool sample of 301 children. It was also re-administered after 2 weeks to 38 randomly selected respondents, for the purpose of evaluating retest reliability. To evaluate inter-informant reliability, the CBCL was administered to 46 respondents (17 alternative caretakers and 29 fathers) alongside the child’s mother. Generalised linear model was used to measure associations with behavioural/emotional scores. We used structural equation modelling to perform a confirmatory factor analysis to examine the seven-syndrome CBCL structure.

**Results:**

During the first phase we found that most of the items could be adequately translated and easily understood by the participants. The inter-informant agreement for CBCL scores was excellent between the mothers and other caretakers [Pearson’s correlation coefficient (r) = 0.89, p < 0.001] and fathers (r = 0.81; p < 0.001). The test–retest reliability was acceptable (r = 0.76; p < 0.001). The scale internal consistency coefficients were excellent for total problems [Cronbach’s alpha (α) = 0.95] and between good and excellent for most CBCL sub-scales (α = 0.65–0.86). Behavioural/emotional scores were associated with pregnancy complications [adjusted beta coefficient (β) = 0.44 (95 % CI, 0.07–0.81)] and adverse perinatal events [β = 0.61 (95 % CI, 0.09–1.13)] suggesting discriminant validity of the CBCL. Most fit indices for the seven-syndrome CBCL structure were within acceptable range, being <0.09 for root mean squared error of approximation and >0.90 for Tucker–Lewis Index and Comparative Fit Index.

**Conclusion:**

The CBCL has good psychometric properties and the seven-syndrome structure fits well with the Kenyan preschool children suggesting it can be used to assess behavioural/emotional problems in this rural area.

## Background

Behavioural/emotional problems are common in children, and externalising behavioural problems such as attention deficit hyperactivity disorder occur in up to 10 % of preschool children [1]. It is difficult to identify these behavioural/emotional disorders in very young children since these children are developing rapidly, and there are few child psychologists or psychiatrists, particularly in resource-poor settings [2]. Nonetheless, the past decade has seen increased focus on diagnosis and description of behavioural/emotional problems in very young children using screening tools that have simpler items, and which can reliably identify behavioural/emotional problems with excellent sensitivity and specificity.

The child behaviour checklist (CBCL) is one such tool which was originally developed in the USA under the auspice of Achenbach System of Empirically Based Assessment (ASEBA) [3]. While the CBCL is applicable for children aged between 1.5 and 5.5 years [3], the preschool Strengths and Difficulties Questionnaire and Rutter Child Behaviour Problem scales are not extended to children under 2 or 3 years of age [4, 5]. The CBCL has been validated in 23 other societies some from low and middle-income countries such as Kosovo, Taiwan and Turkey, where it has shown good psychometric properties [6]. In this landmark study, the CBCL identified behavioural/emotional problems in preschool children with a high sensitivity and specificity (>90 %) compared to a psychiatrists diagnosis [6]. In these validation studies, factor analysis demonstrated that the 100 items of the CBCL measures seven CBCL components which correlate well with Diagnostic and Statistical Manual of Mental Disorders (DSM)-IV syndromes, based upon experts’ evaluations [7]. The CBCL also discriminates children at risk of medical conditions such as epilepsy compared to those not at risk of the condition, underlining its discriminant validity [3]. However, none of these studies were conducted in Africa, where risk factors for neuropsychiatric conditions are common [8, 9].

We have documented behavioural/emotional problems in 26 % of 110 community controls aged 6–9 years selected for an epilepsy study in Kilifi, Kenya [10]. However, psychopathology in older children cannot be generalised to very young children [11, 12]. Infections with a neurological involvement such as malaria are important causes of admissions to Kilifi County Hospital (the main district level referral hospital in this area [8]); and these may be important risk factors for mental health illnesses and behavioural disorders in children. To date no behavioural/emotional studies have been conducted in preschool children in Kenya, largely because of a lack of appropriate tools for this group of children. There are no data in Africa on the reliability of the preschool CBCL in assessing behavioural/emotional problems, but the school-age CBCL was adapted for use in Uganda and was found to be reliable [13].

We examined the psychometric properties of the CBCL in a community sample of preschool children living on the Kenyan coast to compare its performance with that in other countries. We investigated the applicability of the 7-syndrome CBCL structure in these preschool children. We further developed CBCL score ranges that can be used in epidemiological and intervention studies within rural Kenya.

## Methods

### Study site and population

This pilot study was conducted in Kilifi Heath and Demographic and Surveillance System (KHDSS) of the KEMRI-Wellcome Trust Research Programme (http://www.kemri-wellcome.org/index.php/en/study_page/16), which is located on the Kenyan coast. Majority of the people in this area are subsistence farmers and a few fishermen. Literacy level is low and almost 66 % of the population live below the poverty line i.e. live on less than a dollar a day. There is a high prevalence of neurological impairments and epilepsy in children [14].

### Translation of CBCL into local languages

We used a systematic approach of translation and adaptation. The initial translation was done by two independent translators fluent in the original language (English) and the target language (Kiswahili). These translations were then back translated into English by two independent translators. The third step involved evaluation of the translation by a panel of five people fluent in Kiswahili, including two authors of this paper (SK and AA). We conducted focused group discussions and in-depth interviews involving 90 parents and teachers of children with epilepsy (in whom behavioural problems are common) to elicit phrases and idioms to be used in the translated version; most of the CBCL items were perceived as problems that occur in their children [15].

The agreed version was tested in the community with 50 mothers (who were not among the 90 parents who participated in the focused group discussions) to seek participants were requested to provide feedback for each item. The feedback from participants (largely on item wording) was collated and used to create the next version of the questionnaire. Following this evaluation the questionnaire was tested again to ensure that the language used was understandable to the community members. The last stage involved back-translation from Kiswahili into English by an experienced linguist. The back translated version was evaluated by one of the authors (EM, a psychologist) for consistency of meaning with the original CBCL. The few issues raised through this process were resolved through consensus across all the groups involved in the translation process. Our translation process indicated that with adequate consultation it was possible to achieve semantic equivalence; however we did find that literacy levels of participants presented a methodological challenge.

The CBCL was originally designed to be a written questionnaire, however, with the low literacy levels in our population and restricted reading culture, most of our parents cannot fill in the questionnaires themselves. Consequently, a trained fieldworker read out the behaviour problem items to the respondents and documented the respondents rating of the child’s behaviour. An additional problem consistently observed was with the use of a Likert rating scale. To simplify the procedure and enhance accuracy in our population we performed a two stage approach. Firstly we asked if the child had a problem; if the answer was yes we then asked about its frequency or severity to enable a score of 1 or 2.

A signed permission to translate the CBCL was obtained from the developers of the tool (ASEBA) from the University of Vermont’s Research Centre for Children, Youth and Families, Inc.; a non-profit Corporation (Appendix: licence #912-10-21-2013). Our translation was shared with ASEBA, who used it to update an earlier translation.

### Sample size determination

Our sample size determination was based on the principle that alpha coefficients are the most widely used measure for internal consistency in neuropsychological studies and that an adequate sample should be one that produces stable sample coefficient alpha, which provides a precise estimate of the population coefficient alpha [16]. Since sample alpha coefficient is dependent on the first largest *eigenvalue* from principal component analysis (PCA) on the dataset, we estimated that a sample size of at least 100 preschool children will be associated with *eigenvalues* of ≥6 according to a simulation study that utilised a Monte-Carlo method [17], and therefore a sample size of 301 preschool children available in our study would provide unbiased estimator of coefficient alpha.

### Administration of CBCL

The CBCL was administered to 301 parents (mothers, fathers and/or caretakers) of children aged 1–6 years residing within the KHDSS, in the initial phase of the pilot study. The study participants were randomly selected from the KHDSS census database. Based on the multiple caregiving practice in Kilifi we asked the mother to nominate another person who knows the child well to have them respond to the CBCL; 29 alternative caregivers were used in this sub-study and these data were used to evaluate inter-informant reliability. Similarly, 17 mother-father dyads were also interviewed. For test–retest reliability we administered the CBCL to 38 randomly selected respondents after 2 weeks following the initial administration.

### Ethics, consent and permissions

This study was approved by the Kenyan National Ethical Review Committee (SSC No 2599) and parents or caretakers of all children gave written informed consent to participate.

The data used in this study are part of the neurodevelopmental studies at KEMRI-Wellcome Trust Research Programme http://www.kemri-wellcome.org/index.php/en/researcharea/26 and can be to any scientist wishing to use them for non-commercial purposes upon request from the authors.

### Statistical analysis

The data was analysed using STATA (Version 11). Student *t* test or Mann–Whitney test (where appropriate) was used to compare the behavioural/emotional scores between sexes. Generalised linear model of the Gaussian family and with an identity link was used to measure associations between log-transformed behavioural/emotional scores and pregnancy/birth or socioeconomic information or medical factors. Cohen’s kappa coefficients determined the inter-informant agreements between the mother and either fathers or other caretakers for children with behavioural/emotional problems, defined as those with scores ≥90th percentile, considered as the cut off for severe or abnormal CBCL total scores [3]. The test and retest reliability of the before and after assessments was investigated using pairwise correlation coefficients. Cronbach’s alpha was used to evaluate reliability coefficients of the items for the entire tool and for the specific 7-syndrome subscales. The item reliability coefficients first used data from all children, and then for boys and girls separately. Confirmatory factor analysis was used to test the fit index of the 7-syndrome model described by ASEBA in this rural population, using structural equation modelling; which provides standardised factor loading coefficients, and goodness of fit statistics such as root mean squared error of approximation (RMSEA), Comparative Fit Index (CFI) and Tucker–Lewis Index (TLI). The confirmatory factor analysis was done using raw CBCL scores. RMSEA was considered the primary fit index because it performed more robustly in a Monte-Carlo simulation study [18]; while CFI and TLI were considered secondary. Models with modest data fit were modified by allowing correlation of error terms with the largest modification indices (>10) to improve goodness of fit statistics. The cut for acceptable fit indices was ≤ 0.09 for RMSEA and ≥0.90 for CFI and TLI [19].

Internalising scores were formed from emotionally reactive, anxiously depressed, withdrawn and somatic complaints subscales of the CBCL [3]. Externalising scores were derived from attention problems and aggressive behaviour subscales of the CBCL.

## Results

### General description

The CBCL was administered to 301 parents and/or caretakers of preschool children. The 301 respondents comprised of 224 (74.1 %) mothers, 23 fathers (7.6 %) 54 other caregivers (17.9 %). Of the 301 children in the study, 161 (53.5 %) were males. The overall median age was 29 months [interquartile range (IQR), 10–52], with no differences between males and females (p = 0.827).

School attendance was reported in 85/301 (28 %) children. Pregnancy and birth information could be recalled by 185 mothers of whom 22 (12 %) reported pregnancy problems and 10 (5 %) perinatal complications. Socioeconomic and sociodemographic data showed that 116/301 (39 %) mothers were educated, while 118/301 (39 %) mothers were employed. Employment was more common in educated mothers [74/116 (64 %)] than in uneducated mothers [44/185 (24 %)]; p < 0.001. Seizures were diagnosed by a clinician in 17/204 (8 %) children who were invited to come to our clinic for diagnostic evaluation.

### CBCL median scores

**Fig. 1:**
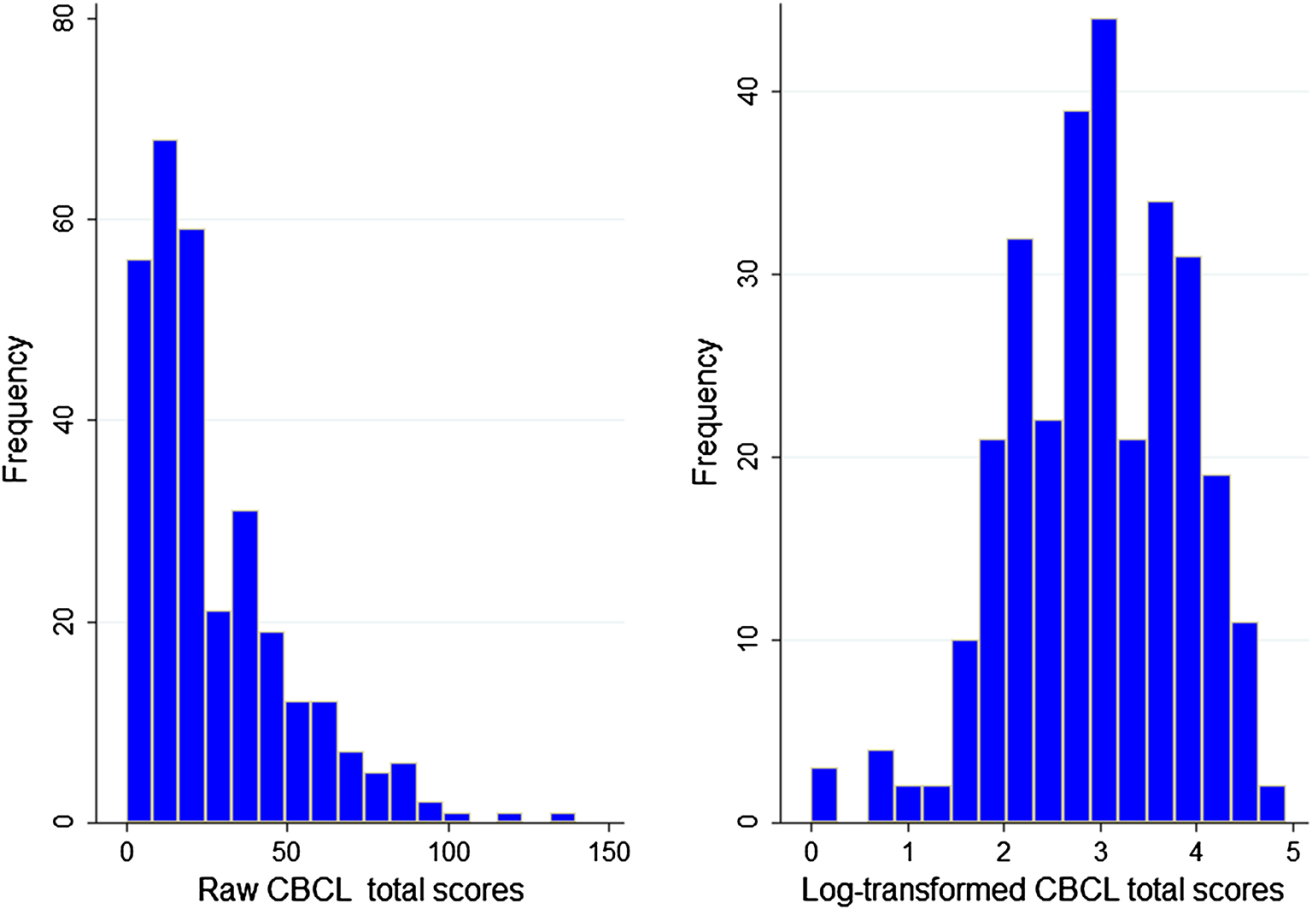
Distribution of raw and log-transformed CBCL scores for 301 preschool children. The raw behavioural scores were skewed to the left and were thus log-transformed to achieve a normal/parametric distribution

The median raw CBCL Total problems scores for all items was 20 (IQR 10–38) and were similar between males and females (p = 0.730). The 90th percentile raw Total problems score was 60 (95 % CI, 52–69). The median raw CBCL score for internalising subscales was 7 (IQR 3–14) while that for externalising subscales the median score was 6 (IQR 3–12). The median raw externalising scores were similar in males and females [6 (IQR 3–11) vs. 6 (IQR 3–14); Z = −0.12, p = 0.898], and so were raw internalising scores [7 (IQR 3–12) vs. 7 (IQR 4–15); Z = 1.01; p = 0.312]. The mean scores for the specific CBCL subscales are shown in Table 1. The raw CBCL total scores were skewed to the left and were therefore log-transformed to achieve a Gaussian distribution for further regression analysis. The distribution of raw and log-transformed CBCL total scores are shown in Fig. 1.

**Table 1:** Median CBCL scores by subscales and sex

Subscales	Scores for all children (IQR)	Scores for boys (IQR)	Scores for girls (IQR)	P value*
Emotionally reactive	1.0 (0–2.0)	1.0 (0–2.0)	1.0 (0–3.0)	0.286
Anxiously depressed	2.0 (0–5.0)	2.0 (0–5.0)	2.0 (0–5.0)	0.419
Somatic complaints	2.0 (0–4.0)	2.0 (0–3.0)	2.0 (0–4.0)	0.363
Withdrawn	2.0 (0–3.0)	1.0 (0–2.0)	2.0 (0–3.0)	0.198
Sleep problems	2.0 (0–3.0)	2.0 (0–3.0)	2.0 (0–3.0)	0.841
Attention problems	2.0 (1.0–4.0)	2.0 (1.0–4.0)	2.0 (1.0–4.0)	0.453
Aggressive behaviour	4.0 (1.0–9.0)	4.0 (1.0–9.0)	4.0 (1.0–9.0)	0.992
Internalising subscales	7.0 (3.0–14.0)	7.0 (3.0–12.0)	7.0 (4.0–15)	0.312
Externalising subscales	6.0 (3.0–12.0)	6.0 (3.0–11.0)	6.0 (3.0–14.0)	0.898

* Mann–Whitney U test

### Associations of pregnancy/birth, socioeconomic and medical factors with behavioural/emotional scores

In a linear regression model accounted for age and sex, only pregnancy complications [β = 0.44 (95 % CI, 0.07–0.81); p = 0.021] and adverse perinatal events [β = 0.61 (95 % CI, 0.09–1.13); p = 0.023] showed a significant association with behavioural/emotional scores. Maternal education [β = 0.15 (95 % CI, −0.10, 0.40); p = 0.233], employment [β = 0.16 (95 % CI, −09, 0.41); p = 216] and history of seizures [β = 0.26 (95 % CI, −0.16, 0.68); p = 0.223] were not associated with behavioural/emotional scores.

### Test–retest reliability

Of the 301 children who were initially assessed with the CBCL, 38 were assessed again after at least two weeks. The initial median CBCL Total problems score for these 38 children was 9 (IQR 7–17), and remained similar with scores after 2 weeks [8 (IQR 6–11)]. The before and after CBCL scores were significantly correlated [Pearson correlation coefficient (r) = 0.76; p < 0.0001].

### Inter-informant agreement

For 17 children, the CBCL was administered to both mothers and the alternative caretaker. There was an excellent inter-informant agreement between the CBCL scores for the mother and those for the caretaker (r = 0.89; p < 0.0001). For 29 children, the CBCL was administered to both mothers and fathers. The inter-informant agreement between the mother’s and father’s CBCL scores was excellent too (r = 0.81; p < 0.0001).

### Internal consistency

The internal consistency of the CBCL as measured by Cronbach alpha was 0.95 (95 % CI, 0.93–0.97) and was 0.95 (95 % CI, 0.94–0.96) for boys and 0.94 (95 % CI, 0.92–0.96) for girls. All the subscales of the CBCL had acceptable to excellent Cronbach’s coefficient alphas (0.65–0.86), except for the withdrawn subscale (0.53) and attention problem subscale (0.57) (Table 2). The Cronbach coefficient alpha was 0.86 (95 % CI, 0.84–0.88) for externalising scores and 0.87 (95 % CI, 0.85–0.89) for internalising scores. The Cronbach’s coefficient alpha for males (0.95) appeared higher than those for females (0.93).

**Table 2:** Scale reliability coefficients for CBCL item scales and goodness of fit statistics for CBCL seven-syndrome structure

Subscales	Cronbach’s alpha: all children (95 % CI)	Cronbach’s alpha: boys (95 % CI)	Cronbach’s alpha: girls (95 % CI)	RMSEA: all children	CFI: all children	TLI: all children
Emotionally reactive	0.70 (0.65–0.75)	0.71 (0.64–0.78)	0.68 (0.61–0.75)	0.039	0.97	0.96
Anxiously depressed	0.74 (0.70–0.77)	0.77 (0.72–0.82)	0.69 (0.62–0.76)	0.050	0.97	0.95
Somatic complaints	0.69 (0.65–0.73)	0.67 (0.61–0.73)	0.71 (0.65–0.77)	0.054	0.94	0.92
Withdrawn	0.53 (0.46–0.59)	0.50 (0.40–0.60)	0.55 (0.45–0.65)	0.000	1.00	1.00
Sleep problems	0.65 (0.60–0.70)	0.72 (0.67–0.77)	0.49 (0.37–0.61)	0.061	0.97	0.93
Attention problems	0.57 (0.50–0.64)	0.59 (0.50–0.68)	0.57 (0.47–0.67)	0.000	1.00	1.00
Aggressive behaviour	0.86 (0.84–0.88)	0.87 (0.85–0.89)	0.84 (0.80–0.88)	0.077	0.83	0.80
Internalising subscales	0.87 (0.85–0.89)	0.87 (0.85–0.89)	0.87 (0.84–0.90)	0.030	0.97	0.95
Externalising subscales	0.86 (0.84–0.88)	0.88 (0.85–0.91)	0.85 (0.81–0.89)	0.039	0.92	0.90

Acceptable coefficient alpha were those >60, while acceptable fit indices were those <0.09 for RMSEA and those >0.90 for CFI and TLI

*CI* confidence interval, *RMSEA* root mean squared error of approximation, *CFI* Comparative fit index, *TLI* Tucker–lewis index

### Standard coefficients and fit indices of the seven-syndrome CBCL structure

All of seven-syndromes of the CBCL reached the mean acceptable cut-off standardised item loadings of 0.35, with “withdrawn” having the lowest at 0.38 (Table 3), although it was still within the ranges reported previously (Table 4) [3]. All the RMSEA, CFI and TLI for the seven-syndrome CBCL structure reached acceptable fit levels, except aggressive behaviours which were slightly below the cut-off (Table 2).

**Table 3:** Standardised item loading coefficients for child behaviour checklist in a Kenyan preschool community sample

Syndrome items	Standardised item loading coefficients (95 % CI)
Emotionally reactive	Overall: 0.47 (0.36–0.58)
Disturbed by any change in routine	0.35 (0.23–0.47)
Nervous movements or twitching	0.33 (0.21–0.45)
Shows panic for no good reason	0.58 (0.48–0.68)
Rapid shifts between sadness and excitement	0.23 (0.11–0.36)
Sudden changes in mood or feelings	0.53 (0.42–0.63)
Sulks a lot	0.63 (0.54–0.72)
Upset by new people or situations	0.42 (0.30–0.53)
Whining	0.59 (0.49–0.68)
Worries	0.60 (0.50–0.70)
Anxious depressed	Overall: 0.53 (0.43–0.63)
Clings to adults or too dependent	0.44 (0.32–0.55)
Feelings are easily hurt	0.53 (0.43–0.63)
Gets too upset when separated from parents	0.50 (0.40–0.61)
Looks unhappy without good reason	0.68 (0.60–0.77)
Nervous, high-strung, or tense	0.51 (0.41–0.59)
Self-conscious or easily embarrassed	0.41 (0.29–0.52)
Too fearful or anxious	0.59 (0.50–0.69)
Unhappy, sad, or depressed	0.58 (0.48–0.68)
Somatic complaints	Overall: 0.46 (0.35–0.57)
Aches or pains (without medical cause)	0.35 (0.24–0.48)
Can’t stand having things out of place	0.31 (0.19–0.43)
Constipated, doesn’t move bowels (when not sick)	0.51 (0.41–0.62)
Diarrhoea or loose bowels (when not sick)	0.59 (0.49–0.68)
Doesn’t eat well	0.27 (0.15–0.39)
Headaches (without medical cause)	0.63 (0.54–0.72)
Nausea, feels sick (without medical cause)	0.58 (0.48–0.68)
Painful bowel movements (without medical cause)	0.49 (0.37–0.58)
Stomach-aches or cramps (without medical cause)	0.70 (0.62–0.78)
Too concerned with neatness or cleanliness	0.20 (0.07–0.32)
Vomiting, throwing up (without medical cause)	0.44 (0.33–0.55)
Withdrawn	Overall: 0.38 (0.28–0.52)
Acts too young for age	0.04 (0.00–0.18)
Avoids looking other in the eye	0.42 (0.28–0.55)
Doesn’t answer when people talk to him or her	0.38 (0.24–0.51)
Refuses to play active games	0.32 (0.18–0.45)
Seems unresponsive to affection	0.47 (0.33–0.61)
Shows little affection toward people	0.58 (0.44–0.73)
Shows little interest in things around her	0.48 (0.33–0.63)
Withdrawn, doesn’t get involved with others	0.32 (0.17–0.47)
Sleep problems	Overall: 0.51 (0.40–0.61)
Doesn’t want to sleep alone	0.22 (0.09–0.34)
Has trouble getting to sleep	0.48 (0.37–0.59)
Nightmares	0.51 (0.40–0.62)
Resists going to bed at night	0.47 (0.36–0.58)
Sleeps less than most kids during and/or night	0.49 (0.37–0.60)
Talks or cries out in sleep	0.70 (0.61–0.80)
Wakes up often at night	0.67 (0.57–0.76)
Attention problems	Overall: 0.45 (0.31–0.60)
Can’t concentrate, can’t pay attention for long	0.59 (0.46–0.73)
Can’t sit still, restless, or hyperactive	0.62 (0.48–0.76)
Poorly coordinated or clumsy	0.39 (0.24–0.54)
Quickly shifts from one activity to another	0.41 (0.27–0.55)
Wanders away	0.26 (0.11–0.40)
Aggressive behaviour	Overall: 0.50 (0.40–0.59)
Can’t stand waiting; wants everything now	0.53 (0.44–0.62)
Defiant	0.52 (0.43–0.62)
Demands must be met immediately	0.52 (0.43–0.61)
Destroys things belonging to his/her family or other children	0.59 (0.51–0.68)
Disobedient	0.36 (0.25–0.47)
Doesn’t seem to feel guilty after misbehaving	0.49 (0.39–0.59)
Easily frustrated	0.51 (0.42–0.61)
Gets in many fights	0.62 (0.54–0.70)
Hits others	0.67 (0.60–0.74)
Hurts animals or people without meaning to	0.20 (0.08–0.31)
Angry moods	0.62 (0.54–0.70)
Physically attacks people	0.53 (0.44–0.63)
Punishment doesn’t change his/her behaviour	0.30 (0.19–0.42)
Screams a lot	0.61 (0.53–0.69)
Selfish or won’t share	0.54 (0.44–0.63)
Stubborn, sullen or irritable	0.60 (0.51–0.68)
Temper tantrums or hot temper	0.52 (0.43–0.61)
Uncooperative	0.27 (0.15–0.49)
Wants a lot of attention	0.38 (0.27–0.49)

Standardised item loading computed with confirmatory factor analysis implemented with structural equation modelling. Individual item loadings were averaged to produce mean loadings for a specific syndrome. Acceptable factor loadings were those >0.40 for the overall subscale

**Table 4:** Comparison of the seven-syndrome correlated CFA model of this present study with ranges from Achenbach and Rescorla, 2000

Syndrome	Items	Mean loadings: present study	Range of mean loadings: Achenbach and Rescorla
Emotionally reactive	9	0.47	0.33–0.73
Anxious depressed	8	0.53	0.21–0.76
Somatic complaints	11	0.46	0.38–0.96
Withdrawn	8	0.38	0.28–0.86
Sleep problems	7	0.51	0.44–0.76
Attention problems	5	0.45	0.39–0.59
Aggressive behaviours	19	0.50	0.16–0.79

## Discussion

This study aimed to examine the utility and validity of the CBCL in assessing behavioural/emotional problems in a rural Kenyan preschool sample. After translation and slight adaptation of the CBCL, overall internal consistency properties were excellent, the test–retest correlation coefficients were good, and the inter-informant agreements with mothers were acceptable for other close caretakers, as well as for fathers. Additionally, most factor loadings and fit statistics for the seven-syndrome CBCL structure were acceptable, establishing the use of these behavioural/emotional constructs in this population.

### CBCL scores and cut-off ranges

The mean CBCL scores (27) in this sample is comparable to 33 from an American sample [3], but lower than those in a Taiwanese (42) [20] and Chinese sample (45); although the latter included adopted children who may have more psychopathology than in the general population [21]. Parents may have underreported the extent of behaviour/emotional problems considering the stigma associated with mental health illnesses [22], particularly as this was the first psychopathology survey of preschool children in this area. Behavioural/emotional scores were similar between sexes and between externalising and internalising scales, consistent with some previous studies [3, 21], but not others [20].

The cut-off CBCL scores for use in epidemiological and intervention studies based on the 90th percentile as recommended by Achenbach and Rescorla [3] is comparable to those of 50–65 reported in other countries [3, 20]. This cut-off score likely represents those at risk of severe behavioural/emotional problems rather than a clinical diagnosis of mental health problems since it is derived from a random rather than a normative sample. The high behavioural/emotional scores in our study are consistent with a high prevalence of neuropsychiatric conditions in this area [14]; the prevalence of behavioural/emotional problems may be higher than the 8–15 % reported in most studies from high income countries [1].

### Associations for discriminant validity

Behavioural/emotional scores were associated with pregnancy complications and adverse perinatal events, supporting the discriminant validity of the CBCL in differentiating at-risk children from those not at risk [3]. No significant associations were observed with seizures and socioeconomic information, but this may be explained by the smaller number screening for seizures, for example. Nonetheless, all these factors investigated should be accounted in associations with behavioural/emotional scores since they can be potential confounders. The CBCL may therefore be used by clinicians to identify children at risk of behavioural/emotional problems, following medical conditions or early life exposures, who would benefit from behavioural/emotional interventions.

### Test retest and inter-informer reliability

The good test–retest reliability scores asserts the stability of the CBCL in assessing behaviour over time, although psychopathology can change in developing children [23]. Our test–retest reliability was better than that reported from a Luganda version of the CBCL (0.76 vs. 0.67), but the Uganda study used the school-aged CBCL [13]. Inter-informant agreement was acceptable for both fathers and caretakers, although the former was lower than the latter; which is similar to UK studies using the Strengths and Difficulties Questionnaire [24]. Indeed in anecdotal reports from the field team a number of fathers noted that they were not very familiar with their children’s behavioural/emotional patterns. On the contrary, caretakers such as grandmothers, stepmothers and/or aunts showed good inter-informant agreement with the mothers; as they spend more time caring for these children.

### Internal consistency

All empirically-based seven-syndromes, as defined by ASEBA [3], were associated with acceptable to excellent reliability coefficient alphas, underscoring the value of the CBCL in assessing behavioural patterns in this Kenyan rural population. A Luganda version of the school-aged CBCL had good reliability coefficient alpha (0.83) [13], which is slightly lower than in our preschool CBCL (0.95). Total problem coefficient alpha of 0.95 is highly similar with those documented in the USA (0.95) [3], China (0.93) [21], and Taiwan (0.95) [20]. The coefficient alpha for “withdrawn” and “attention problems” were slightly lower than in other studies [3, 20, 21], perhaps because in this population emotional behaviours are considered less serious than disruptive behaviours. This finding may suggest that some items describing withdrawn and aggressive behaviours are understood differently in Kenya than in the USA.

### Seven-syndrome structure and fit indices

Our Confirmatory Factor Analysis, implemented with structural equation modelling, supported the seven-syndrome CBCL structure, whose fit indices were acceptable. In particular, the standardised factor loadings are comparable to the ranges provided by Achenbach and Rescorla who first validated the CBCL in the USA [3]. The slightly smaller loadings in a few items in our study (withdrawn and attention problems) are in part explained by performing polychoric (for 3-point response scales) rather than tetrachoric (for 2-point response scales) item correlations; the former is deemed appropriate for the CBCL but may be associated with lower factor loadings [18]. The few items with very low standardised coefficients may have been misunderstood by parents and should be investigated further in future studies before they omitted from future assessments using CBCL to examine behavioural/emotional problems in Kenyan populations. All RMSEA and most CFI and TLI indices suggested an acceptable to good fit for the seven-syndrome CBCL structure in our population. In particular, our overall RMSEA of 0.035 is better than the 0.06 from the USA [3], 0.053 from China [6], 0.055 in Taiwan [20] and up to 0.059 from 23 other societies [6], probably because we allowed item error terms to correlate [19]. These findings support configural invariance of the CBCL and its application across diverse societies, including rural Kenya. Since the internal structure of the CBCL in this population is satisfactory, future studies can evaluate other properties, in particular the predictive validity as these children grow older [11].

### Strengths and limitations

The strength of this study is the careful translation of the CBCL into the local languages and use of trained and experienced field assistants to administer the tool. Training of fieldworkers by one psychologist and comparison of their scoring for concordance before collection of the CBCL data helped avoid introduction of inter-rater bias. The sample size was acceptable to run confirmatory factor analysis and to determine overall internal consistency. The sample size may however have been small for some sub-analysis. Withdrawn and attention problems scales were associated with low internal consistency. Test–retest reliability and interinformant agreement were not performed for subscales of the CBCL, since these scales had low scores which were skewed, and these factors would overinflate the correlation coefficients. The derived cut-off score doesn’t represent a clinical diagnosis of a mental health problem since it is based on a random rather than a normative sample.

## Conclusion

A culturally and contextually adapted CBCL possesses good to excellent psychometric properties and has acceptable fit indices for the seven-syndrome structure; and thus can be used to assess behaviour in preschool children in this rural area of Kenya. However, these findings should be validated in other African settings since cultural and socioeconomic differences may exist which can influence behavioural assessments and outcomes. Future studies should develop clinical cut-offs for behavioural/emotional problems based on normative samples of children without neuropsychiatric problems, and examine the predictive validity of the CBCL when these children grow older. Epidemiological studies to estimate reliable estimates of psychopathology in this area are justified to inform the development of appropriate behavioural interventions.

## Abbreviations

ASEBA: Achenbach System of Empirically-Based Assessments
CBCL: Child Behaviour Checklist
DSM: Diagnostic and Statistical Manual of Mental Disorders
CFI: Comparative Fit Index
TLI: Tucker Lewis Index
RMSEA: Root Mean Squared Error of Approximation
